# Deep Learnability: Using Neural Networks to Quantify Language Similarity and Learnability

**DOI:** 10.3389/frai.2020.00043

**Published:** 2020-06-24

**Authors:** Clara Cohen, Catherine F. Higham, Syed Waqar Nabi

**Affiliations:** ^1^English Language & Linguistics, University of Glasgow, Glasgow, United Kingdom; ^2^School of Computing Science, University of Glasgow, Glasgow, United Kingdom

**Keywords:** second-language acquisition, deep learning, language similarity, computational modeling, artificial languages

## Abstract

Learning a second language (L2) usually progresses faster if a learner's L2 is similar to their first language (L1). Yet global similarity between languages is difficult to quantify, obscuring its precise effect on learnability. Further, the combinatorial explosion of possible L1 and L2 language pairs, combined with the difficulty of controlling for idiosyncratic differences across language pairs and language learners, limits the generalizability of the experimental approach. In this study, we present a different approach, employing artificial languages, and artificial learners. We built a set of five artificial languages whose underlying grammars and vocabulary were manipulated to ensure a known degree of similarity between each pair of languages. We next built a series of neural network models for each language, and sequentially trained them on pairs of languages. These models thus represented L1 speakers learning L2s. By observing the change in activity of the cells between the L1-speaker model and the L2-learner model, we estimated how much change was needed for the model to learn the new language. We then compared the change for each L1/L2 bilingual model to the underlying similarity across each language pair. The results showed that this approach can not only recover the facilitative effect of similarity on L2 acquisition, but can also offer new insights into the differential effects across different domains of similarity. These findings serve as a proof of concept for a generalizable approach that can be applied to natural languages.

## 1. Introduction

Learning a second language (L2) can be difficult for a variety of reasons. Pedagogical context (Tagarelli et al., 2016), cognitive processing differences across learners (Ellis, 1996; Yalçın and Spada, 2016), L2 structural complexity (Pallotti, 2015; Yalçın and Spada, 2016; Housen et al., 2019), or similarity between the target L2 and the learner's first language (L1) can all conspire to affect the speed and success of L2 acquisition (Hyltenstam, 1977; Lowie and Verspoor, 2004; Foucart and Frenck-Mestre, 2011; Málek, 2013; Schepens et al., 2013; Türker, 2016; Carrasco-Ortíz et al., 2017). Similarity, in particular, is a difficult variable to examine, because it is so hard to pin down. Although individual structures (e.g., relative clauses, cognate inventory) can be compared fairly straightforwardly across languages, it is much harder to combine these structures appropriately to determine a global similarity metric across languages, which limits our ability to predict how difficult an arbitrary L2 will be to acquire for different L1 speakers. The goal of this paper is to propose a new method for evaluating the effect of similarity on the learnability of L2 structures, using deep learning.

Current approaches to determining similarity effects on L2 acquisition typically take an experimental angle, usually proceeding in one of two ways. The first is to take one group of L2 learners, with the same L1, and compare their acquisition of different structures in the L2, such that one structure is similar to L1 and the other is different. For example, Lowie and Verspoor (2004) observed that Dutch learners of English acquire prepositions that are similar in form and meaning across the two languages (e.g., *by/bij*) more easily than ones that are dissimilar (e.g., *among/tussen*). Foucart and Frenck-Mestre (2011) found tentative evidence German learners of French show more electrophysiological sensitivity to gender errors when the French nouns have the same gender as German than when they have a different gender. This observation was later supported by Carrasco-Ortíz et al. (2017), who observed a similar pattern with Spanish learners of French. Díaz et al. (2016) examined Spanish learners of Basque, and found stronger electrophysiological responses to syntactic violations in structures that are common between Spanish and Basque compared to violations in structures unique to Basque. Türker (2016) found that English learners of Korean performed better at comprehending figurative language when the expressions shared lexical and conceptual structure across the two languages than when they diverged. Overall, then, it seems that at the lexical, morphosyntactic, syntactic, and conceptual levels, learners have an easier time acquiring L2 structures that are similar to the L1 equivalents than structures that are different.

The second type of experimental approach holds constant the target structures to be learned, and instead compares the acquisition of those structures across learners with different L1s. Málek (2013), for example, found that Afrikaans-speaking learners of English acquired prepositions, which divide up the conceptual space in very similar ways in the two languages, better than Northern Sotho speakers, which treats those same meanings quite differently. Kaltsa et al. (2019) found that German-Greek bilingual children, whose L1s have a gender system similar to Greek's, performed better on gender agreement tasks than English-Greek bilingual children, whose L1 has no such gender system. In a very large-scale study, Schepens et al. (2013) found that Dutch learners had more difficulty acquiring Dutch when their native languages' morphological systems were less similar to Dutch—especially if that dissimilarity lay in a reduced complexity. This approach, too, shows that similarity between L1 and L2 seems to aid learning.

Although these findings all agree that L1/L2 similarity is important, they nevertheless all rely on binary same/different evaluations at a feature by feature level. Yet even if language grammars could be neatly decomposed into binary feature bundles, it's not at all clear whether those features are equally strong in determining similarity. Are two languages more similar if they share relative clause construction, for example, or subject-verb agreement patterns? And even if we can arrive at a hierarchy of feature strength within a domain, it's not at all clear how similarity can be compared across domains. Is a language pair more similar if both employ a particular conceptual organization of spatial relations, or if both rely on suffixing concatenative morphology? And if a pair of languages share similar syntactic patterns but utterly distinct morphology, are they more or less similar than a pair of languages that share morphological structure, but are utterly dissimilar in syntax? Even if we assume that all linguists work from the same theoretical underpinnings when characterizing grammatical structures, these questions make it clear that using feature-by-feature comparisons to characterize linguistic similarity has severe limitations.

**Figure 1:**
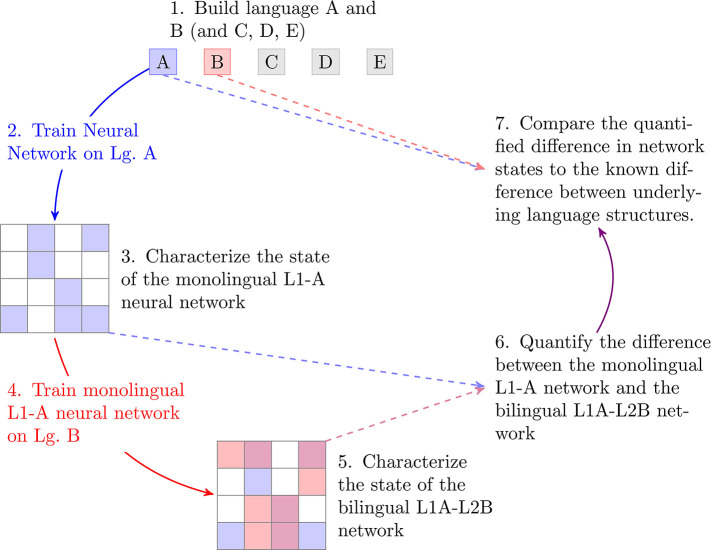
Summary of the process for generating learnability metrics across artificial language pairs.

To avoid these problems, our approach employs deep learning, using changes in neural network activity before and after learning a second language as a proxy for the learnability of that language. In the work presented here, we restrict ourselves to carefully controlled artificial languages, as a proof of concept, but the approach is scalable to natural languages. Our process, illustrated in Figure 1, starts with a set of five artificial languages, whose similarity across pairs was systematically controlled (Step 1). Next, we trained Long Short Term Memory (LSTM) neural networks on each of these five languages, producing five models representing monolingual L1 speakers for each of the five languages (Step 2). After characterizing the state of these monolingual networks (Step 3) we then retrained them on a second language (Step 4), crossing each possible L1 with each possible L2, to create a set of 20 “bilingual” networks. Twenty such networks are possible because there are 10 possible combinations of the five languages, and each combination counts twice—once for a network with an L1 of language A learning an L2 of language B, and then again for the reverse. We then characterized the state of each bilingual network (Step 5), and quantified the change of state that had to take place during the L2-acquisition process (Step 6). This change of state represents the “learnability” of each L2 for a speaker of each L1. Finally, we compared these learnability metrics to the built-in degree of similarity across different artificial language pairs (Step 7).

If our approach can capture the findings from experimental research in a scalable manner, then networks should show less change when learning L2s that are similar to the known L1. Further, by controlling the domains of similarity between the languages (e.g., morphology vs. syntax), we can examine which types of similarity are most effective in aiding L2 acquisition.

## 2. Methods

### 2.1. Artificial Languages

We built five artificial languages—Alpha, Bravo, Charlie, Delta, and Echo—which could vary across three dimensions: vocabulary, morphology, and syntax. We built multiple versions of each linguistic subsystem: two vocabularies, labeled A and B; three morphologies, labeled C, D, and E, and two syntactic systems, labeled F and G. By distributing the different linguistic subsystems across the five languages, we were able to manipulate language similarity systematically.

Table 1 illustrates this manipulation. For example, Alpha has vocabulary A, morphology C, and syntax F. Charlie has vocabulary B, morphology E, and syntax G. It therefore shares none of these properties with Alpha, which makes the two languages maximally dissimilar. Alpha and Charlie therefore appear in the left-most column of Table 2, under the heading “No overlap.” By contrast Bravo has vocabulary A, morphology D, and syntax G, which means it overlaps with Alpha in exactly one dimension—vocabulary—while differing in morphological and syntactic systems. Alpha and Bravo therefore appear in the middle column, under the heading “One dimension > Morphology.” Alpha and Delta overlap in two dimensions—sharing vocabulary A and Syntax F—and so that language pair appears in the rightmost column, under the heading “Two dimensions > Vocabulary/Syntax.” This distribution of features ensured that every combination of domain and degree of similarity was represented: One language pair was maximally dissimilar, sharing neither vocabulary, morphology, nor syntax; five language pairs overlapped in one of those three features; and four language pairs overlapped in two features.

**Table 1:** Summary of the degree of overlap (zero/one/two dimensions), and domains of similarity (vocabulary/morphology/syntax) across the ten possible language pairs.

**No overlap**	**One dimension**	**Two dimensions**
alpha/charlie (ACF/BEG)	*Vocabulary*	*Vocabulary/Morphology*
	alpha/bravo (ACF/ADG)	delta/echo (AEF/AEG)
	alpha/echo (ACF/AEG)	*Vocabulary/Syntax*
	bravo/delta (ADG/AEF)	alpha/delta (ACF/AEF)
	*Morphology*	bravo/echo (ADG/AEG)
	charlie/delta (BEG/AEF)	*Morphology/Syntax*
	* Syntax*	charlie/echo (BEG/AEG)
	bravo/charlie (ADG/BEG)	

**Table 2:** Layers of the model employed here.

**Layer**	**Description**	**Output activations**	**Learnable weights**
Sequence input	Input one word at a time	1	N/A
Word embedding	Each input word in the training data vocabulary is mapped to 100 features	100	100 word embedding activations × NV
LSTM	Each input of 100 word embedding features is mapped to 100 features in output. This layer contains several input, recurrent, and bias gates which learn to retain or forget input features.	100	There are twelve types of weights [4 input (100 × 100), 4 recurrent (100 × 100) and 4 bias (100 × 1)].
Dropout	To prevent overfitting, 20% of the input to this layer is masked, and hence dropped	100	N/A
Fully connected	The input (100 activations) are each mapped to the training data vocabulary	NV	NV × 100 + NV bias
SoftMax	Transforms the input distribution of weighted training data vocabulary into a probability distribution, aka wordscore	NV	
Classification	The wordscore is sampled to determine the identity of the next word	1 word	

*NV refers to the number of words in the training data vocabulary–here, 330. Note that the out from each layer serves as the input to the following layer*.

For each vocabulary, we constructed a set of 330 word roots, divided into six different lexical categories: nouns, verbs, adjectives, determiners, prepositions, and conjunctions. In each vocabulary, there were 100 of each of the three different classes of content words (nouns, verbs, adjectives) and 10 of each of the three different classes of function words (prepositions, determiners, conjunctions). For the sake of simplicity, we used the same set of phonotactic rules to generate the words in each vocabulary, but we ensured that there was no overlap between the two lexicons. This ensured identical phonotactic systems across all languages. Nevertheless, since the neural networks used here did not look below the level of the morpheme when learning each language, the similarity in phonotactics across the two vocabularies was not able to affect the learning process in this work.

The three morphological systems are all concatenative, but the features that appear and the types of morphosyntactic processes varied across each language. For example, morphology C had suffixing number concord in NPs, such that a plural suffix appeared on determiners, nouns, and adjectives, while singular NPs were unmarked. Plural subject NPs conditioned plural agreement suffixes on the verb, while tense markers for past, present or future appeared on verbs as prefixes. By contrast, morphology D had prefixing number on nouns and adjectives, but determiners had no number marking. Rather, determiners conditioned definiteness agreement on nouns, such that a lexically specified set of definite determiners conditioned a definite suffix on nouns, while the indefinite determiner conditioned an indefinite suffix. Verbs showed no agreement, but prepositions assigned either accusative or dative case on NP complements.

The two syntactic systems varied primarily in whether they were head-initial (Syntax F) or head-final (Syntax G). Sentences could be one clause or two clauses; clauses could contain subjects and verb phrases; and verb phrases could contain direct objects and prepositional phrases. However, for the sake of simplicity we did not allow any recursion: Sentences could not extend beyond two clauses, and noun phrases could not be modified by prepositional phrases.

Examples (1–2) below illustrate the type of sentence generated in Alpha and Charlie, respectively, the maximally different languages. The “translation” below each example provides an English sentence with similar syntactic structure to the two sentences^1^. Note that the meanings of the individual words in the English glosses are arbitrary, as we did not build any semantic content into these languages.

(1) Alpha:
  *piaux-s toutsheosh-s dishkeof will-piaufsizh-s kagh kiaug* those-PL dog-PL large FUT-run-PL in the.SG
  diaushziauv gatdux
  garden-SG empty
  “Those large dogs will run in the empty garden.”
(2) Charlie:
  *us-biaus koutbux pakpuz-himherthem dud-one doutghouf* ACC-the empty garden-ACC in-SG run
  heshethey-dag teopbous buxxouf
  NOM-this large dog
  “This large dog will run in the empty garden.”

### 2.2. Model Building

#### 2.2.1. Training Data

For each language, we generated 200,000 sentences by randomly selecting phrase structure rules in a top-down walk through the language's grammar. For example, if the language could contain single-clause or dual-clause sentences, we would randomly select a single clause sentence. A single clause sentences required a noun phrase subject, so the walk moved down to the noun phrase structure rule. Given the option of a singular or plural subject noun phrase, we would randomly select a plural noun phrase, and given the option of an adjective modifier or not, we would randomly select no adjective. This process was repeated for all syntactic structures in the sentence, right down to the vocabulary selection. The process was repeated to generate 200,000 sentences, which were filtered to remove any repetitions.

#### 2.2.2. Training the Monolingual Model

A wide variety of neural network model structures have been developed for use with linguistic data (for an excellent overview of their use with both natural and artificial language, see Baroni, 2019). We trained a long short-term memory (LSTM) model on our generated sentences. During training, this model learns the parameters which define layers in the network model through optimization of a loss function. These layers are essentially maps that take input features and map them to output features. Thus, across the different layers these features or activations represent language at different levels of abstraction. After training, the models are able to generate new text word-by-word for each language they had been trained on.

As Table 2 shows, there are many different layers included in the full model. However, the key layers for our purposes were the word embedding layer and especially the LSTM layer. The word-embedding layer has learnable weights and maps input to 100 output features. At the end of training, each word in the vocabulary was associated with a unique vector of word embedding feature weights, which allows the model to uncover internal regularities in the vocabulary, such as part of speech, or grammatical definiteness. We refer to these features simply as “word embeddings,” and conceptualize them, roughly, as the ability of the model to learn the lexicon of a new language.

The LSTM layer also has learnable weights associated with memory gates and 100 hidden cells; it takes output from previous layers as its own input, and maps it to 100 features. Each hidden cell is associated with a set of 12 learnable parameters, which control how much information about the input is retained or forgotten during training and generation. Once these weights are learned, the features are inputed into the final layers to create probability distributions for the next possible word, given a preceding word sequence. The next word actually generated by the model is the result of sampling from that probability distribution. These hidden cells can be roughly conceptualized as the ability of the model to learn the grammar of a new language, including the dependencies within and across sentences^2^.

During training, the model learns the parameters which define the layers through optimization of a loss function, using stochastic gradient descent (Hochreiter and Schmidhuber, 1997). Each language model saw the training data five times, and the amount of data for each language was kept constant at 200,000 sentences across all the models. The execution platform used was the Deep Learning Toolbox from MATLAB (MATLAB, 2019).

The entire training process took about fifteen minutes on a GPU, or about two hours on a CPU. All training data, code, and output models are available on our OSF archive: https://osf.io/6dv7p/?view_only=4575499b2daf473fbd6a04ca49213218. Readme files are included to allow the reader to run the code on their own machine, but we also provide trained nets for the users to download and analyse to complement our own analysis below.

#### 2.2.3. Evaluating the Monolingual Model

**Figure 2:**
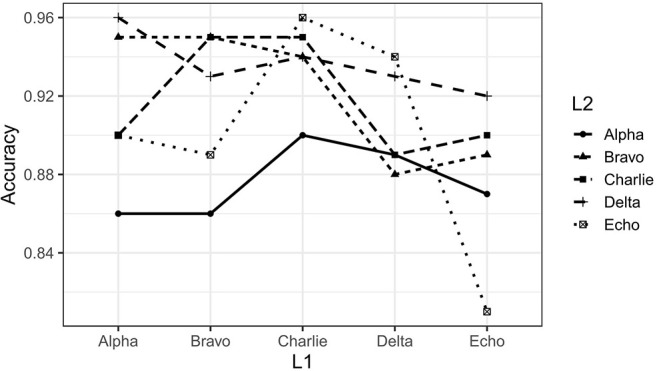
Summary of parsing accuracy for monolingual and bilingual models. Lines connect the results for outputs produced in the same target language. Datapoints where L1 and L2 are the same represent the monolingual models; datapoints where L1 and L2 are not the same represent bilingual models.

After training was completed, we evaluated the success of language learning by having the model generate 100 sentences, and then running those sentences through the Lark automatic parser (https://github.com/lark-parser/lark) to see whether they could be parsed according to the grammar that was used to generate the original training data. On average our monolingual models were able to produce fully parsable sentences about 90% of the time. The lowest accuracy rate was 81% (Echo), and the highest was 95% (Bravo and Charlie. See Figure 2 for the full set of accuracy rates. Monolingual models are those points for which L1 and L2 are the same language).

#### 2.2.4. Training the Bilingual Model

To create our “bilingual” models, we took our monolingual models, and retrained them on data from each of the other four languages. Thus, our monolingual model trained on Alpha was retrained on data from Bravo, Charlie, Delta, and Echo, producing four bilingual models, each with the same L1 but a different L2. This process was repeated for each monolingual model, resulting in five monolingual models, and twenty bilingual models representing each possible combination of L1 and L2. The parsing accuracy of bilingual models was on average 91%, ranging from a low of 86% (L1-Bravo, L2-Alpha) to a high of 96% (L1-Alpha, L2-Delta; and L1-Charlie, L2-Echo). These accuracy rates are given in Figure 2.

With our trained monolingual and bilingual models in hand, we are now prepared to address the key research question: How does similarity between L1 and L2 across different domains of linguistic structure affect learnability in neural network models?

### 2.3. Characterizing Learnability Using Neural Networks

#### 2.3.1. Output-Oriented Approaches

There are three ways we see for determining how learnable a second language is for our models. The first is to examine the output of the trained models, to determine the percentage of sentences that are grammatically correct. Although we did examine the accuracy of our model outputs using the Lark parser (see above), we do not consider it a useful measure beyond a rough check that learning has occurred. First, this approach scores each sentence as a binary parsing success or failure. In other words, the parser would treat word salad—or indeed, alphabet soup—as exactly the same sort of failure that would result from a misplaced agreement suffix in an otherwise flawless sentence. Yet if we were to try to determine some degree of “partial credit” parsing, to reward correct phrases in ungrammatical sentences, we would need to make certain theory-dependent decisions. A complete verb phrase, for example, can be as simple as a single verb, but it can also include direct object noun phrases and prepositional phrase adjuncts. What should be done in the case of a well-constructed verb phrase that nevertheless contains an ungrammatical direct object embedded within it—and is an ungrammatical noun phrase argument a worse violation than an ungrammatical prepositional phrase adjunct? If so, what about an ungrammatical noun phrase that is the argument of a preposition, but the prepositional phrase is itself merely an adjunct to the verb phrase? These decisions will need to be informed by theoretical assumptions of syntactic and morphological structure, and to the extent that they are theory-dependent, they are not objective measures of learning.

A second approach to assessing learnability is to look at the training time required to learn the second language. The model's progress could be monitored during training, and the learnability could be measured in terms of the amount of time needed to reach a particular success criterion. Yet what should count as the success criterion? The default learning curve in our models tracks the accuracy with which the model predicts the next word in the training data as a function of what it has already seen, but the ability to predict the next word in a training sentence is very different from the ability to generate novel sentences that respect the underlying structural patterns in the training data. In principle the model could be asked to generate sample sentences after each training epoch to track its progress learning those patterns, but then those sentences would need to be scored for accuracy, which brings us back to the problem of partial credit vs. binary parsing as described above.

In principle, these problems are not insurmountable. Yet even if we had well-motivated, theory-independent ways to score the model output for accuracy, these approaches neglect a fundamental strength of using neural networks to study second-language learnability—a strength that goes well beyond the scalability and generalizability of computer simulations. This strength is the ability to examine the internal structure of a model. We cannot open up the heads of bilingual learners and examine the behavior of their specific language-learning neurons as they produce each individual word or sentence in their second language. But with neural networks, we can.

#### 2.3.2. Network-Structure Approach

Our network-structure approach essentially asks how much the underlying structures of the network models themselves must change in order to learn a new language. Further, by asking whether the amount of change varies depending on the types of words or sentences being produced, we can also pinpoint the domain in which a language is more or less learnable. The less a network must change in order to learn a new language, or the less it must change to produce a structure in that new language, the more learnable that language or structure is.

To characterize the network structures, we made each model generate 100 sentences, and recorded the activation of each of the 100 network cells for each word in each sentence. We then calculated each cell's mean activation for each part of speech by averaging across all 100 sentences. Because not all languages shared the same inventory of morphological prefixes and suffixes, we extracted only the root parts of speech that were constant across all languages—namely, adjectives, conjunctions, determiners, nouns, prepositions, verbs, and End Of Sentence, which we treated as a word type of its own. Finally, for each cell in each part of speech, we subtracted its mean activation in L2 from its mean activation in L1, and took the absolute value of that difference. If a cell's mean activation changed greatly in the process of learning L2, then the absolute difference will be high; by contrast, if it retained roughly the same activation pattern in L1 and L2, then the absolute difference in mean activation across the 100 sentences will be minimal.

## 3. Results

We predicted that the model would need to change less to learn L2s that were similar to L1, than L2s that were different from L1. For each language pair, we coded the amount of overlap as 0, 1, or 2. Thus, our L1-Alpha, L2-Charlie model had an overlap of 0, as did our L1-Charlie, L2-Alpha model. The two models pairing Alpha and Bravo had an overlap of 1, and Alpha and Delta had an overlap of 2 (see Table 1).

In addition to coding language pairs by degrees of overlap, we also coded each them for the domain of overlap, to explore whether that affected the amount of cell activity change produced by learning a second language. For example, the language pairs Alpha and Bravo, along with Charlie and Delta, both have an overlap degree of 1, but Alpha and Bravo overlap in vocabulary, while Charlie and Delta overlap in morphology (see Table 1).

We analyzed degree and domain of overlap separately, because domain of overlap perfectly predicts degree of overlap.

### 3.1. Degree of Overlap

Degree of overlap refers to the number of linguistic domains (vocabulary, morphology, syntax) that are shared between two languages (see Table 1). We analyzed the effect of degree of overlap on mean activity change with mixed effects linear regression, using the R programming environment (version 3.6.1), with the package lme4 (version 1.1–21). We set the absolute mean activity difference between L1 and L2 as the dependent variable, and for independent variables we included L1, L2, part of speech, and overlap degree. Random effects included random intercepts for each cell^3^.

The model estimates supported our initial predictions. Cell activity differences were lower for languages with one degree of overlap than for languages with no overlap [β = −0.023, *SE* (β) = 0.012, *t* = −1.91], and also for languages that overlapped by two degrees [β = −0.031, *SE* (β) = 0.014, *t* = −2.28].

**Figure 3:**
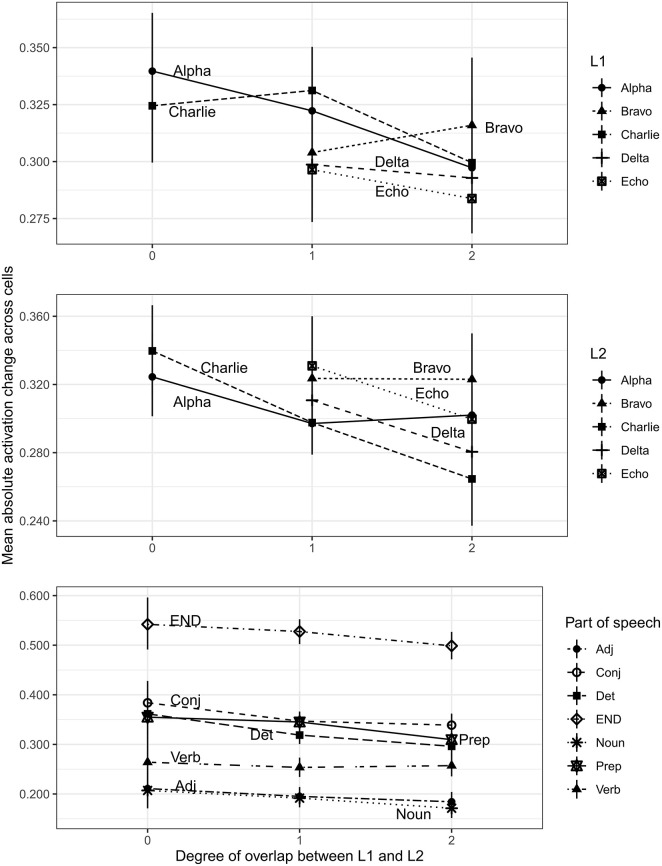
Absolute cell activity change for models learning L2 after L1, averaged across all 100 hidden cells. The top panel shows the activity change grouped by the L1; the middle panel shows the activity change grouped by the L2; and the bottom panel shows the activity change when grouped by part of speech.

Not every language participated in pairings for all three degrees of overlap, because Bravo, Delta, and Echo did not have any partners in which there was no overlap. As a result, L1 and L2 would have produced a rank-deficient contrast matrix if we attempted to put an interaction between these terms and overlap degree in the model. Nevertheless, we can see apparent language-specific variation in the effects of overlap in the top two panels of Figure 3.

Figure 3 shows the absolute mean activity change between L1 and L2, averaged across all cells for the twenty possible L1/L2 language pairings. The x-axis indicates the degree of overlap between the two languages, which can range from 0 (no overlap; maximally dissimilar) to 2 (two domains of overlap; maximally similar). In the top panel, the twenty languages are grouped according to the starting point—the L1 that the model learned before it was retrained on an L2. In the middle panel, they are grouped according to the L2; and in the bottom panel they are grouped according to the particular part of speech.

In both of the top two panels, we can see that Bravo is the one language that bucks the pattern of reduced activity change with higher degrees of overlap. Learners with an L1 of Bravo (top panel) increase their activity change when learning L2s of two degrees of similarity compared to L2s with one degree of similarity; and when learners are learning Bravo as an L2, they show no difference in activity change regardless of whether Bravo overlaps with their L1 by one or two degrees. Nevertheless, all the other languages show a decrease in cell activity change as L1/L2 similarity increases.

The lowest panel in Figure 3 collapses across L1s and L2s, and instead shows the changes in cell activity that are necessary to produce each part of speech in the L2. Interestingly, these grammatical distinctions show distinct clusters. Nouns and adjectives do not require much change in cell activity, while verbs condition a bit more. The function words—conjunctions, determiners, and prepositions—on the other hand, cluster together, appreciably above the content words. The largest change in cell activity that is necessary is associated with the end of the sentence: cells must learn distinctly different activation patterns in order to know when to stop an utterance than they must learn to produce words within that utterance.

All of these observations emerged in the regression model. Compared to adjectives, nouns were not significantly different [β = −0.007, *SE* (β) = 0.01, *t* = −0.746], while all other parts of speech showed significantly greater changes in cell activity (all *t*s>5). Relevelling our factors revealed that function words showed significantly more activation change than verbs [conjunctions β = 0.091, *SE* (β) = 0.01, *t* = 9.26; determiners β = −0.058, *SE* (β) = 0.01, *t* = 5.87; prepositions β = −0.076, *SE* (β) = 0.01, *t* = 7.69]; and among function words, conjunctions showed significantly more activation change than determiners [β = 0.033, *SE* (β) = 0.01, *t* = 3.38], although not prepositions [β =.015, *SE* (β) = 0.01, *t* = 1.57]. Determiners did not differ significantly from prepositions [β = −0.018, *SE* (β) = 0.01, *t* = −1.82].

### 3.2. Domain of Overlap

To analyse the effects of domains of overlap (e.g., syntax vs. morphology for language pairs with one degree of overlap, or syntax/morphology vs. vocab/morphology for language pairs with two degrees of overlap), we built a mixed effects linear regression model, with the same software as in our degree analysis. Again we set the absolute mean activity change as the dependent variable, and used part of speech and overlap domain as independent variables, and cell identifier as random intercepts.

**Figure 4:**
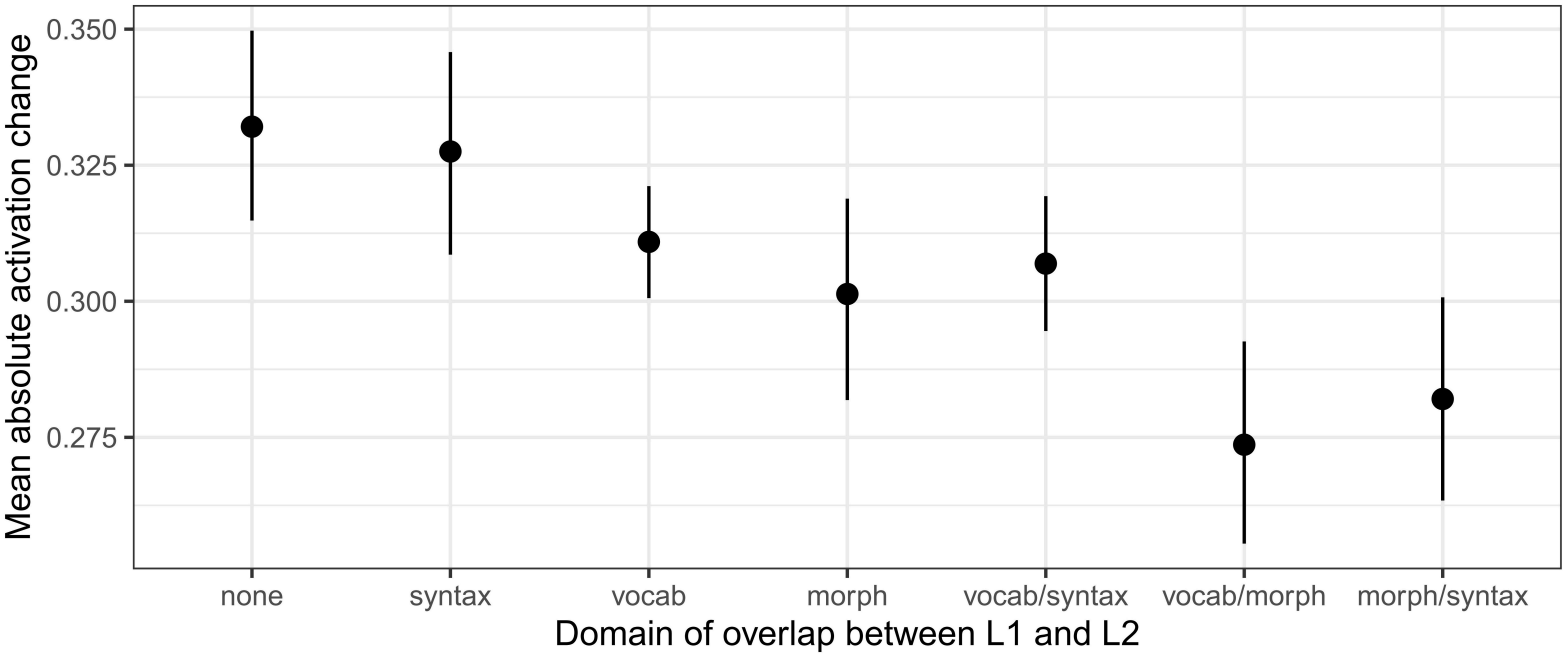
Absolute cell activity change for models learning L2 after L1, averaged across all 100 hidden cells, organized by the domain in which language pairs overlapped.

Two key patterns that emerged in the model are visible in Figure 4. First, shared syntax does little to reduce cell activity differences between L1 and L2. Language pairs which overlap only in syntax showed no significant difference in cell activation change from language pairs which do not overlap at all [β = −0.004, *SE* (β) = 0.013, *t* = −0.36]. Further, language pairs which overlap in vocabulary and syntax show similar reductions in cell activity as language pairs which overlap in vocabulary alone [vocabulary alone: β = −0.021, *SE* (β) = 0.010, *t* = −2.06; vocabulary and syntax: β = −0.025, *SE* (β) = 0.011, *t* = −2.31]. Second, shared morphology seems to be the most helpful in reducing changes in cell activity between L1 and L2—especially when it is combined with a second degree of overlap. Languages which overlap in morphology alone show lower differences in cell activity than languages with no overlap [β = −0.031, *SE* (β) = 0.013, *t* = −2.44], or which overlap only in syntax; and languages which overlap in morphology and some other domain have the lowest differences in cell activation [morphology and vocabulary: β = −0.058, *SE* (β) = 0.013, *t* = −4.64; morphology and syntax: β = −0.050, *SE* (β) = 0.013, *t* = −3.97].

## 4. Discussion

This project used artificial language learners and artificial languages to test a method of investigating L2 acquisition that has considerable potential for expansion. By building artificial languages, we were able to sidestep the problem of defining how similar two languages are, because we could hard-code into the artificial languages a known degree of overlap. By measuring the changes in neural networks that had been trained on these languages, we were able to estimate the learnability of a language by focusing not on output, but on changes within the generative machine itself. Our results supported our predictions: More degrees of overlap between an L1 and L2 led to less change in network activity. In other words, more similar languages were easier to learn.

### 4.1. Key Domains and Structures for Learning

Our results are particularly intriguing because they offer insights into which components of linguistic similarity, and which linguistic structures, seem to require the most learning during second language acquisition. We observed that a shared morphological system between L1 and L2 in particular seemed to result in easier learning; while shared syntactic structures seemed to make very little difference. Further, function words (conjunctions, determiners, and prepositions) seemed to be harder to learn compared to content words (adjectives, and nouns, although to a lesser extent verbs).

Both of these patterns may reflect the way in which linguistic dependencies are encoded in these artificial languages. Because our syntactic grammars were fairly simple, dependencies such as subject-verb agreement, or number concord in noun phrases, were largely expressed through morphological affixes. Syntactic structures were actually quite similar: all sentences needed subjects and verbs; all verb phrases could be transitive or intransitive, with optional prepositional phrase adjuncts; and all sentences could have one or two clauses, with the latter combining the clauses through the use of a conjunction. Although the linear order in which these structures were combined varied, the nature of the dependencies was quite similar. By contrast, the morphological systems could vary widely: with different features—tense, number, definiteness—expressed or ignored depending on which morphology a language had. This fundamental similarity across syntactic systems, compared to a wider degree of variability in morphological systems, may explain why shared morphology proved more useful in learning a new language than shared syntax.

To the extent that the syntax of these languages *did* encode dependencies, however, it largely was encoded in the function words. Determiners were obligatory in noun phrases; conjunctions were required in two-clause sentences; and prepositions required noun phrase objects. Adjectives, by contrast, were entirely optional; and nouns were often optional in verb phrases, because verbs could be either transitive or intransitive. This could explain why so much more of the network activity changes emerged in the production of content words than on function words, as shown in the bottom panel of Figure 3.

If this account is accurate, it can explain why, among content words, verbs were harder to learn than nouns and adjectives. Verbs were often the locus of agreement morphology, as well as tense inflection; and unlike adjectives and nouns, verbs' appearance was most restricted: in each clause they were obligatory, and also limited to exactly one appearance per clause. Yet this limitation was also shared across all the languages. As a result, verbs required more learning than nouns and adjectives, but less than the more structurally complex content words.

### 4.2. Potential for Generalization

Because deep learning packages are sophisticated enough to learn natural languages as well as artificial languages (Sutskever et al., 2011; Graves, 2013, for an excellent recent overview, see Baroni, 2019), we believe that this approach can be generalized to natural languages, and allow researchers in language acquisition to make testable predictions about how learnable second languages might be for speakers of different first languages. Although there is a robust pedagogical tradition for certain language pairs (e.g., Spanish for English speakers, or French for German speakers), these resources are limited to dominant language groups which provide a large population of L1 learners, or which are popular L2 target languages. For such learners, existing pedagogical approaches are nuanced and mature. Yet for speakers of Finnish, who wish to learn Malayalam or Quechua, there may be very few resources that are targeted to their existing knowledge.

Naturally, it will be necessary to apply these methods to natural languages to see whether the patterns that we found in a sterile simulation generalize in any meaningful way. Our current analysis, for example, did not consider the role of phonological similarity, although research in bilingualism has shown that the phonological structures of L1 and L2 can interact in complex ways. For example, substantial similarity between L1 and L2 phonologies may actually interfere with the development of distinct L2 phoneme categories (Flege, 1995, 2007). This pattern which may well pose a challenge for our results, which show generally facilitative effects of similarity. On the other hand, we did not ask our models to learn the phonology of the languages we constructed, and so we cannot know whether they would replicate natural language findings of inhibitory effects of phonological similarity, or mis-predict facilitatory effects.

We also did not consider the role of semantics in our artificial languages, which rendered it impossible to explore or model the effects of cognates (words with similar forms and meanings in two languages) or false friends (words with similar forms and dissimilar meanings) as a domain of language similarity. Yet these types of lexico-semantic relationships have been shown to affect both language learning (Otwinowska-Kasztelanic, 2009; Otwinowska and Szewczyk, 2019), as well as bilingual processing (van Hell and Dijkstra, 2002; Duyck et al., 2007; Brenders et al., 2011). Further, these effects interact in complex ways not only with properties of the broader linguistic context, but also individual cognitive properties of the speaker (Schwartz and Kroll, 2006; van Hell and Tanner, 2012; Dijkstra et al., 2015). Our models could in principle be adjusted to reflect different speaker properties—e.g., more or fewer hidden cells or word embeddings could perhaps model differences in working memory capacity—but we did not manipulate those properties here. All models had identical internal structures, and effectively modeled the identical person in multiple learning situations.

Our findings should not be overstated, but we do believe they serve as a persuasive proof of concept of our methods: Given a known set of underlying relationships between L1 and L2, our modeling procedure can uncover them in ways that are linguistically meaningful. Yet interpreting the output of this approach when it is applied to natural languages will not be straightforward, and will need to be guided by the already robust psycholinguistic literature on bilingual language processing and acquisition. Nevertheless, we are optimistic. Our approach, while still in its early stages, has the potential to democratize language learning, by predicting not only which languages are easier to learn for which speakers, but also of identifying which domains of grammar may be most challenging.

## Data Availability Statement

The grammars, training data, and code used in this study can be found in the Open Science Framework repository here: https://osf.io/6dv7p/?view_only=4575499b2daf473fbd6a04ca49213218.

## Author Contributions

CC led the project and the analysis of results, assisted in developing the artificial languages, and wrote the majority of the article. CH handled the neural network models and assisted in analysing the results. SN assisted in developing the artificial languages, analysing the results, and managing project logistics. Both CH and SN assisted in article revisions.

## Conflict of Interest

The authors declare that the research was conducted in the absence of any commercial or financial relationships that could be construed as a potential conflict of interest.

## Footnotes

^1^Of course, the morphological systems are quite different from English, and cannot be translated “accurately.” In 2, for example, the verb is translated as future tense for comparability with the explicit future tense in 1, but since Charlie has no tense marking on verbs, this could just as easily have been past tense.

^2^We did not include cross-sentence or cross-clausal dependencies in our grammars. However, natural languages have many of them, ranging from switch-reference conjunctions in Quechua subordinate clauses (Cohen, 2013), to long-distance cross-clausal agreement in Tsez (Polinsky and Potsdam, 2001) to basic pronoun co-reference in English.

^3^Cells were coded according to both their identifier number (1–100) and also for the L1. The L1-coding was necessary because a given cell is only equivalent for every bilingual network whose L1 was the same: Each of those bilingual networks had the same set of L1-trained cells before they learned their distinct L2s. However, if the L1s were not the same, then the cells with matching identifier numbers share no more than a coincidental label, because Cell 1 in a network trained on Alpha as an L1 is utterly distinct from Cell 1 in a network trained on Bravo.
